# Cerebellar Atrophy and Neurocognitive Disorder as Primary Presentation of Antiphospholipid Syndrome in a Young Male

**Published:** 2019-02-26

**Authors:** Sinan Khayyat, Rawaa Ebrahem, Daly Al-Hadeethi, Ammar Al-Obaidi, Shadi Shahouri

**Affiliations:** 1 University of Kansas School of Medicine-Wichita, Department of Internal Medicine; 2 Arthritis and Rheumatology Clinics of Kansas, Wichita, KS

**Keywords:** antiphospholipid syndrome, cerebellar atrophy, neurocognitive disorder, hydroxychloroquine

## INTRODUCTION

Antiphospholipid syndrome (APS) is a multisystem autoimmune disorder characterized by arterial or venous thrombosis and pregnancy morbidity in the presence of antiphospholipid antibodies (aPL).^1^ It can be primary or secondary. Primary antiphospholipid syndrome occurs in the absence of any other related disease. Secondary antiphospholipid syndrome occurs with other autoimmune diseases, such as systemic lupus erythematosus.

The presence of aPL can be demonstrated in one of three ways: the presence of anticardiolipin antibodies (aCL), β 2-glycoprotein I antibodies (GPI), or lupus anticoagulant antibodies (LA).^1^ To meet classification criteria for antiphospholipid syndrome, patients should have one clinical criterion, either vascular thrombosis (venous or arterial) or pregnancy morbidity (at least one fetal death or preterm delivery or three or more unexplained, consecutive, spontaneous pregnancy losses) and one laboratory criterion, the presence of aPL antibodies need to be seen twice and at least 12 weeks apart for confirmation. Neurological manifestations are common in APS and are attributed mainly to vascular thrombosis and aPL-induced neuronal tissue injury. The most common neurological presentation is an ischemic cerebrovascular accident (CVA) or transient ischemic attack (TIA). However, clinical presentations including cognitive dysfunction, headaches, seizures, and psychosis may be atypical in some cases, which makes diagnosis more difficult.^2^

In our case, a male patient initially presented with a neurocognitive disorder, dementia, cerebral atrophy, and seizure of unknown etiology. A diagnosis of APS was made after a brain biopsy revealed microinfarcts and intimal fibrosis and an aPL antibody test was positive.

## CASE REPORT

A 37-year-old male presented to the clinic with an abnormal gait, chronic fatigue, muscle stiffness, and cutaneous bruises. He had suffered these symptoms for many years and initially was diagnosed with a cerebellar neurodegenerative disease of unknown cause. He had seizures and neuropsychiatric symptoms that primarily was represented with mild cognitive impairment and frequent headaches with nonspecific anxiety. Initial brain images revealed atrophy of the brain without a clear explanation.

**Figure 1:**
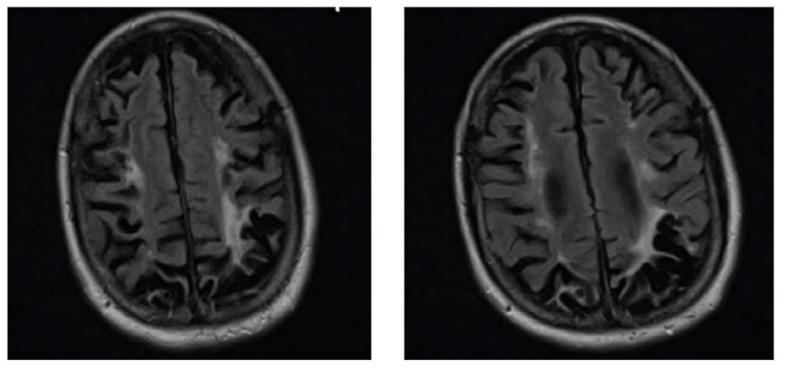
An MRI of the brain showed an increased white matter signal in the centrum semiovale representing encephalomalacia. There was generalized prominence of the cortical sulci representing diffuse cerebral volume loss.

A physical exam showed significant abnormal gait and muscle strength of 4/5 in both the upper and lower extremities. Primary lab results revealed acute kidney injury and thrombocytopenia. Hypercoagulable panel studies (e.g., protein C, protein S, prothrombin gene mutation, and Factor V Leiden) were collected and results were unremarkable. A follow-up MRI showed worsening brain atrophy secondary to chronic cerebral ischemia, and a brain biopsy showed microinfarcts and intimal fibrosis (Figure 1). An underlying autoimmune disease was suspected, and the patient’s work-up came back with the following results: cardiolipin IgG >100 GPL U/ml (negative <10 GPL U/ml, strongly positive ≥80 GPL U/ml), β 2-GP1 IgG >100 U/ml (positive ≥15 U/ml), aPTT 63 S (normal <40 S), and an aPTT-LA mix that remained high.

The patient met the criteria for antiphospholipid syndrome according to the presence of antiphospholipid antibodies in addition to the vascular thrombosis, which led to microinfarctions of the brain microcirculation that eventually resulted in cerebral and cerebellar atrophy and neurocognitive disorder. Treatment was started with aspirin 81 mg daily, hydroxychloroquine 200 mg twice daily, and warfarin with targeted INR (2 – 3). On subsequent visits, the patient reported improvement in his headaches, muscle stiffness, and overall general condition. Most importantly, since starting treatment, surveillance brain imaging has remained stable, and the patient’s renal function has improved.

## DISCUSSION

The incidence of cerebellar CVA is approximately 1.5% with an average age of 62 years.^3^ The main causes are atherosclerosis and cardiac emboli. CVA is relatively rare in younger age groups, and a thorough evaluation to rule out thromboembolic disease should be considered for younger patients. The prevalence of APS with ischemic CVA is as high as 22%, and patients with CVAs are 5.48 times more likely to have the aPL antibodies than the patients without CVAs (95% CI 4.42–6.79).

Neurological manifestations have been reported due to thrombosis, cerebral ischemia, and direct neuronal cell injury by anticardiolipin antibodies, which also can lead to neuropsychiatric symptoms including headache, migraines, chorea, anxiety, and cognitive disorders.^4^,^5^ These neurological manifestations may be confused with other neurological syndromes. The presence of aPL has strong correlations with ischemic CVA, cognitive deficits, and white matter lesions;^6^ furthermore, patients who are β 2-GPI-positive have a twofold increased risk of CVA within 15 years of follow-up compared with aCL-negative individuals.^7^

The guidelines recommend anticoagulation for secondary prevention.^8^ Warfarin is the standard of care with INR of 2 – 3. Ongoing studies examine the efficacy and safety of direct oral anticoagulants in thrombotic APS, especially rivaroxaban, which potentially provides an additional benefit to the anticoagulant effect, by limiting complement activation in APS patients with thrombotic events while they are on warfarin. On the other hand, hydroxychloroquine (HCQ) has been used in SLE patients, but some studies report that HCQ has a very important role in APS patients by decreasing the aPL antibody titers, preventing recurrent arterial thrombosis, and inhibiting platelet aggregation by inhibiting aPL-induced platelet GPIIb/IIIa receptor expression.^9^,^10^

Early treatment with anticoagulants and aspirin in those patients are crucial to prevent recurrent CVAs and TIAs.^9^ HCQ has an important role to decrease the antibody titers and anti-inflammatory effect. Several experimental measures are addressing the immunomodulatory mechanism in treatment of anti-phospholipid syndrome, through inhibition tissue factor upregulation, which is an important mechanism that explains the pro-thrombotic effects of aPL. These measures include tissue factor inhibition, p38 mitogen-activated protein kinase inhibition, nuclear factor-kB inhibition, platelet glycoprotein receptor inhibition, hydroxychloroquine, statins, inhibition of b2GPI and/or anti-b2GPI binding to target cells, complement inhibition, and B cell inhibition. Further studies are required for the role of HCQ in secondary thrombosis prevention.

The treatment of recurrent thrombosis is controversial. However, the available options are targeting high INR, using direct oral anticoagulants, and adding HCQ. The possibility that the INR may not reflect the true anticoagulant intensity in APS patients should be considered. This is due to the variable responsiveness to LA of the reagents used in the INR test, leading to the potential instability of anticoagulation. In our case, the patient started on oral anticoagulants and aspirin to prevent further transient ischemic attacks (TIAs) and CVAs, and that is what kept his brain imaging stable.

## CONCLUSIONS

A high suspicion index and a thorough workup for all patients with CVA presenting before the age of 50 are warranted. Data on etiologies and risk factors for young adults with stroke comes from a few single center or population-based cohorts.^11^–^13^ Vasculopathy (such as arterial dissection), cardiac defects, recent pregnancy, other genetic hypercoaguable states, smoking and illicit drug use, premature atherosclerosis, hypertension, low physical activity, metabolic disorders, and possibly migraine should be on the differential list for such presentations. APS should be added and included in the differential list of diagnoses, especially when the patients present with atypical neurological disorders including cerebellar neurodegenerative disease, seizure, and intractable headache.
